# Resistant starch reduces glycolysis by HK2 and suppresses high-fructose corn syrup-induced colon tumorigenesis

**DOI:** 10.1007/s00535-024-02138-3

**Published:** 2024-08-14

**Authors:** Ying Zhang, Weiyi Shen, Zhehang Chen, Jiamin He, Lijun Feng, Lan Wang, Shujie Chen

**Affiliations:** 1 Department of Gastroenterology, Second Affiliated Hospital of Zhejiang University School of Medicine, Hangzhou, China; 2 Department of Gastroenterology, Sir Run Run Shaw Hospital, Zhejiang University School of Medicine, Hangzhou, China; 3 Department of Pathology, Sir Run Run Shaw Hospital, Zhejiang University School of Medicine, Hangzhou, China; 4 Department of Nutriology, Sir Run Run Shaw Hospital, Zhejiang University School of Medicine, Hangzhou, China

**Keywords:** Colorectal cancer, High-fructose corn syrup, Resistant starch, Gut microbiota, Glycolysis

## Abstract

**Background:**

The intake of high-fructose corn syrup (HFCS) may increase the risk of colorectal cancer (CRC). This study aimed to explore the potential effects and mechanisms of resistant starch (RS) in HFCS-induced colon tumorigenesis.

**Methods:**

The azoxymethane/dextran sodium sulfate (AOM/DSS) and *Apc*^*Min/*+^ mice models were used to investigate the roles of HFCS and RS in CRC in vivo. An immunohistochemistry (IHC) staining analysis was used to detect the expression of proliferation-related proteins in tissues. 16S rRNA sequencing for microbial community, gas chromatography for short-chain fatty acids (SCFAs), and mass spectrometry analysis for glycolysis products in the intestines were performed. Furthermore, lactic acid assay kit was used to detect the glycolysis levels in vitro.

**Results:**

RS suppressed HFCS-induced colon tumorigenesis through reshaping the microbial community. Mechanistically, the alteration of the microbial community after RS supplement increased the levels of intestinal SCFAs, especially butyrate, leading to the suppression of glycolysis and CRC cell proliferation by downregulating HK2.

**Conclusions:**

Our study identified RS as a candidate of protective factors in CRC and may provide a potential target for HFCS-related CRC treatment.

**Supplementary Information:**

The online version contains supplementary material available at 10.1007/s00535-024-02138-3.

## Introduction

Colorectal cancer (CRC) is the third most commonly diagnosed cancer worldwide and the second most frequent cause of death [1]. It is reported that the global burden of cancer is projected to more than double over the next two decades [2], raising the prospect of an enormous public health hazard. Improper diet, nutrition, and physical activity rank high among the most important determinants of human cancer risk [3, 4], particularly CRC [5–7].

High-fructose corn syrup (HFCS), the primary sweetener used in sugar-sweetened beverages (SSBs), is widely used in candy, carbonated drinks, bread, and some other foods [8]. However, with the extensive use of HFCS, its disadvantages have been gradually emerging. Some studies have revealed that the excessive intake of HFCS would increase the risk of metabolic diseases, such as obesity and type 2 diabetes mellitus [9–11]. In addition, Marcus D Goncalves et Al. confirmed that HFCS could enhance intestinal tumor growth in mice in the absence of obesity and metabolic syndrome, and the activation of glycolysis played a vital role in this process [12]. A prospective study also revealed that the high intake of SSBs during adolescence was associated with an increased risk of conventional adenoma, especially rectal adenoma [13]. The intake of HFCS may increase the risk of CRC. However, although its harmfulness is clear, it is impractical to completely prohibit the consumption of HFCS in our daily life. How to reduce or inhibit its carcinogenic effects is an urgent problem to be solved.

Recently, resistant starch (RS), a representative of dietary fiber, has garnered increasing attention from the public and scientific community alike [14]. RS cannot be digested by human amylases in the small intestine and moves into the colon, where it undergoes fermentation by gut microbiota [15]. RS can be classified into four types, type 1 to type 4 according to its properties [16], among which type 2 RS as raw granules has been widely evaluated in animal and human studies [17, 18]. Ingestion of RS could be a promising dietary approach for alleviating chronic kidney disease [19], rheumatoid arthritis [20], systemic lupus erythematosus [21], etc. This approach is supported by the potential mechanisms involving variations in gut microbiota and metabolites [16, 22, 23]. Interestingly, researches revealed that oral supplementation of RS in patients with CRC could inhibit cell proliferation in the upper part of colonic crypts [24] and may reduce colon cancer risk from red meat [25]. However, whether RS could rescue the promotion by HFCS of CRC and its underlying mechanisms remain largely unknown.

In this study, our results revealed that type 2 RS suppressed HFCS-induced colon tumorigenesis in both azoxymethane/dextran sodium sulfate (AOM/DSS) and *Apc*^*Min/*+^ mice models through reshaping the microbial community. Mechanistically, the alteration of microbial community after RS supplement increased the levels of intestinal short-chain fatty acids (SCFAs), especially butyrate, leading to the suppression of glycolysis and CRC cell proliferation by downregulating HK2.

## Methods

### Animal assays

CRC is a life-threatening disease that can be a complication of inflammatory bowel diseases or develop spontaneously, and AOM/DSS and *Apc*^*Min/*+^ mice models were used in our research. C57BL/6 mice were purchased from Shanghai SLAC Laboratory Animal, China. *Apc*^*Min/*+^ mice were purchased from Nanjing Biomedical Research Institute of Nanjing University, China. All mice were maintained in ventilated cages with 12-h light/dark cycles, constant temperature and humidity, enriched water, and ad libitum feeding under SPF conditions. All animal experiments used in this study were approved by the Institutional Animal Care and Use Committee of Zhejiang University.

For inflammation-related carcinogenesis model, C57BL/6 male mice (8 weeks old) were given one single intraperitoneal injection of carcinogen AOM (Sigma-Aldrich, USA) at 10 mg/kg body weight, followed by five successive days of 2% DSS (Sigma-Aldrich, USA) in the drinking water, and then given regular drinking water for 2 weeks. This cycle was then repeated twice. Meanwhile, the three groups were given the following treatments separately: common feed + PBS, common feed + 5% HFCS (45% glucose + 55% fructose), 20% RS (Hi-maize^®^ 260, a commercial type 2 RS supplementation produced from naturally modified high amylose corn [26], Ingredion, USA) + 5% HFCS. PBS and HFCS were administrated by gavage every day (400 μl), while 20% RS was fed freely and mixed with their daily feed. During the modeling process, the disease severity of mice in each group was evaluated by the DAI score, which included the index of weight, stool characteristics, and degree of blood in the stool of mice. After 2 months, mice were killed and the colons were surgically excised for further analysis.

For spontaneous adenoma model, C57BL/6 J *Apc*^*Min/*+^ male mice (3–5 weeks old) were randomly assigned to three groups. The three groups were given different treatments separately, consistent with the grouping in AOM/DSS mice model. At the indicated time intervals, colon and small intestine tissues were harvested after fasting.

In addition, for the subcutaneous tumor model, C57BL/6 mice (4–6 weeks old) were randomly divided into three groups. Drinking water was supplemented with antibiotics cocktail (0.2 g/L ampicillin, neomycin, and metronidazole, and 0.1 g/L vancomycin) for the whole duration of the experiment to deplete the gut microbiota as previously reported [27]. Dietary treatment with either RS or control diet was continuously given to mice through the entire experiment. 5 × 10^6^ MC38 cells were injected subcutaneously into the right flank of C57BL/6 mice (100 μl per mouse). After 5 days of implantation, the tumor volume was monitored every 2 days and calculated as follows: Volume = 0.5 × L × W^2^, where L is the longest diameter and W is the shortest diameter. At the end of the time, mice were killed and the subcutaneous tumors were surgically excised for further analysis.

### Ki67 staining

Colon tumor tissues were fixed in 4% buffered formalin immediately after dissection of mice. The fixed tissues were then dehydrated in ethanol, embedded with paraffin and sectioned at 5 μm. IHC staining analysis of Ki67 was performed as previously described [28]. Tissue microarrays were scanned with a digital slide scanner (Pannoramic MIDI, 3D HISTECH) after staining and processed with Pannoramic viewer software. The intensity of staining in cells was automatically calculated by Quant center software. H-score was acquired according to the formula: H-score = (percentage of weak intensity area × 1) + (percentage of moderate intensity area × 2) + (percentage of strong intensity area × 3).

### 16S rRNA sequencing of the microbial community

Genomic DNAs were extracted from mice colon tissues by QIAamp DNA Mini Kit (Qiagen, Germany) according to the manufacturer’s instructions. 16S rRNA sequencing and bioinformatics analysis were performed by Majorbio BioPharm Technology Company in China. Raw fastq files were demultiplexed and quality filtered by Trimmomatic and merged by FLASH (Fast Length Adjustment of Short Reads to Improve Genome Assemblies). Samples were identified by barcodes and primers, then sequences were dereplicated and discarded. OTUs were clustered with 97% similarity cutoff using UPARSE (version 7.1 http://drive5.com/uparse/). We used Shannon index to measure species richness (α-diversity) of the gut microbiome. β-Diversity of the gut microbiome was calculated using the UniFrac distance between samples and visualized using the principal coordinate analysis (PCoA) (http://www.majorbio.com/).

### Targeted metabolomics of SCFAs

2.5 g metaphosphoric acid was dissolved in 100 ml deionized water, and then 0.6464 g crotonic acid was added to prepare a crotonic acid/metaphosphoric acid solution. The fermentation broth and crotonic acid/metaphosphoric acid solution were evenly mixed and stored at – 40 °C for 24 h. After acidification, samples were centrifuged to separate the supernatants from the precipitate (13,000 r/min, 4 °C) and filtered via a 0.22 μm hydrophilic micron membrane. Then, 150 μl filtered solution was used for gas chromatography. The column temperature heating conditions were: column temperature: 80 °C for 1 min, increased to 190 °C (10 °C per minute), and maintained for 0.5 min; then increased to 240 °C (40 °C per minute) and maintained for 5 min; FID detector: 240 °C; gasification chamber: 240 °C; carrier gas: nitrogen flow rate 20 ml per minute, hydrogen flow rate 40 ml per minute, air flow rate 400 ml per minute. The obtained data were recorded.

### Targeted detection of glycolysis products

Targeted detection of glycolysis products and further analysis were performed by Metware Technology Company. Briefly, colon tumor tissues stored in a – 80 °C refrigerator were thawed and smashed, and then mixed with 70% methanol/water. After different speeds of centrifugation, the supernatant was transferred for further LC–MS analysis. Next, the sample extracts were analyzed using an LC–ESI–MS/MS system (UPLC, ExionLC AD, https://sciex.com.cn/; MS, QTRAP^®^ 6500 + System, https://sciex.com/) and AB 6500 + QTRAP^®^ LC–MS/MS System, equipped with an ESI Turbo Ion-Spray interface, operating in both positive and negative ion modes and controlled by Analyst 1.6 software (AB Sciex). For differential metabolites selected, significantly regulated metabolites between groups were determined by VIP ≥ 1 and absolute Log_2_FC (fold change) ≥ 1.0. The VIP values were extracted from OPLS-DA result, which also contains score plots and permutation plots, and generated using R package MetaboAnalystR. The data was log transformed (log_2_) and mean centered before OPLS-DA.

### Cell culture

Human CRC cell lines (LoVo, HCT116), human normal colonic epithelial cell line NCM460, and mouse CRC cell line MC38 were purchased from American Type Culture Collection (ATCC). HCT116 cultured in Maccoy 5A (Genom, China), LoVo cultured in F-12 K (Genom, China), and NCM460 and MC38 cultured in DMEM medium (GIBCO, China) were supplemented with 10% FBS (Sijiqing, China) at 37 °C in a humidified 5% CO_2_ atmosphere.

### CCK-8 assay

For CCK-8 assay, HCT116, LoVo, or NCM460 cells with the indicated treatment were seeded at 2 × 10^3^ cells per well in 96-well plates, and added with PBS, HFCS, or butyrate plus HFCS in the culture medium. Then CCK-8 assay was performed by the Cell Counting Kit-8 assay kit (Meilunbio, China) according to the manufacturer’s instructions. Briefly, after removing the medium, cells were incubated with CCK-8 for 2 h and the absorbance was determined at 450 nm by a Bio-Rad microplate reader (BioTek, Winooski, VT, USA) at 0, 24, 48, 72, and 96 h, respectively. Each test was repeated five times.

### Lactic acid detection

PBS, HFCS, or butyrate plus HFCS was added to the culture medium of LoVo or HCT116 cells with the indicated treatment separately. After 48 h of treatment, the culture medium was collected for lactic acid detection by lactic acid assay kit (Nanjing Jiancheng Bioengineering Institute, China) according to the manufacturer’s instructions.

### RNA extraction and quantitative RT-PCR

Total RNAs were extracted from CRC cell lines with the indicated treatments using TRIzol reagent (Invitrogen, USA), and cDNAs were reversed by HiScript^®^ II Q RT SuperMix for qPCR (Vazyme, China) according to the manufacturer’s instructions. Quantitative RT-PCR analysis was performed in triplicate in a ROCHE LightCycler480 System (Rotor gene 6000 Software, Sydney, Australia). Each reaction was tested in triplicate in 10 μl reaction system containing ChamQ Universal SYBR qPCR Master Mix (Vazyme, China), primers and template cDNAs. The relative mRNA expression was calculated using the comparative cycle method (2^−ΔΔCt^). β-Actin served as the internal reference genes. The primers are listed in Supplementary Table 1.

### Western blot analysis

Cell extracts were collected using RIPA buffer (Beyotime, China) containing protein phosphatase inhibitor (Solarbio, China) on ice and quantified by BCA Protein Assay Kit (Fdbio science, China). Total proteins were loaded on to 10% polyacrylamide-SDS gels, followed by the transfer of electrophoresed proteins onto the PVDF membranes. The membranes were blocked in 5% BSA (Fdbio science, China) for 2 h and then incubated with primary and secondary antibodies. Subsequently, the signals were detected using an ECL Kit (Fdbio science, China) by ChemiDoc Touch Imaging System (Bio-Rad, USA). The following antibodies were used: HK2 (22029-1-AP, Proteintech), β-actin (AC026, ABclonal), HRP goat anti-rabbit IgG (H + L) (AS014, ABclonal), HRP goat anti-mouse IgG (H + L) (AS003, ABclonal).

### Cell transfection

The siRNAs were transfected into cells using Lipofectamine RNAiMAX (Thermo Fisher Scientific, USA) in opti-MEM (Genom, China) according to the manufacturer’s instructions. The following sequences of siRNAs were used:

siNC: 5ʹ-UUCUCCGAACGUGUCACGUTT-3ʹ;

siHK2-1: 5ʹ-CCAAAGACAUCUCAGACAUUG-3ʹ;

siHK2-2: 5ʹ-CCAGAAGACAUUAGAGCAUCU-3ʹ.

### Plasmid construction and transfection

Plasmid expressing HK2 (NM_000189.5:455-3208) was constructed by GenePharma (Shanghai, China). Transient plasmid transfection was carried out using FuGENE HD transfection reagent (Promega, USA) according to the manufacturer’s instructions.

### Statistical analysis

Statistical analysis was performed using the GraphPad Prism 7.0 software. Data were analyzed with Student’s *t* test, Wilcoxon rank-sum test, or linear regression as shown in figure legends. *P* value less than 0.05 was considered statistically significant.

## Results

### RS suppressed HFCS-induced colon tumorigenesis in AOM/DSS mice

**Fig. 1:**
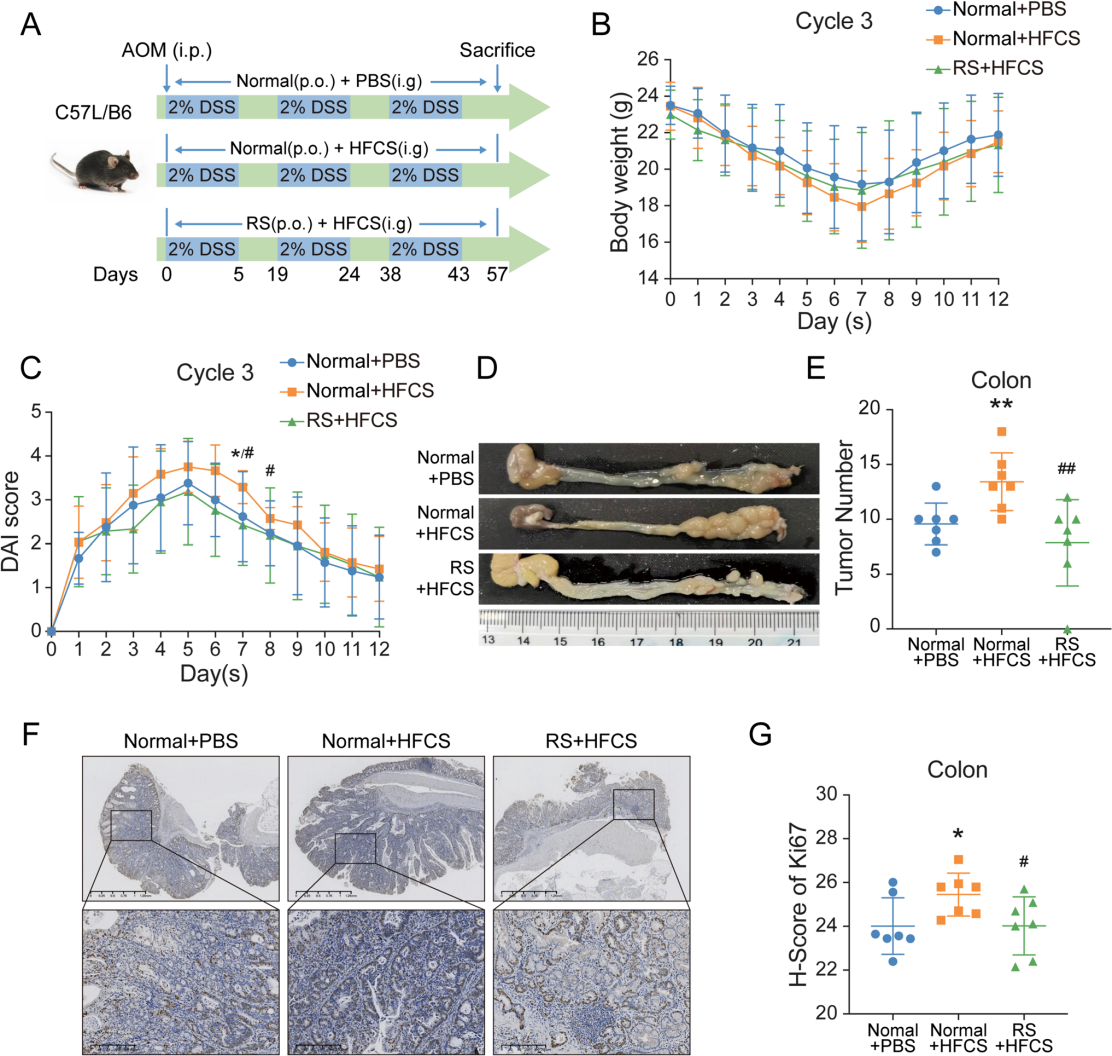
RS suppressed HFCS-induced colon tumorigenesis in AOM/DSS mice. **A** Schematic illustration of AOM/DSS mice model in vivo. **B**, **C** Body weight (**B**) and DAI scores (**C**) of AOM/DSS mice in cycle 3 of DSS administration. **D** Representative colon images of AOM/DSS mice treated with HFCS (*n* = 7), RS + HFCS (*n* = 7), or PBS (*n* = 7) as control. **E** The number of tumors per mice was quantified. **F**, **G** Representative IHC images of Ki67 protein expression in colon tumor tissues from the indicated mice are shown, and the relative expression is quantified by H-score. *The difference between group PBS and group HFCS. #The difference between group HFCS and group HFCS + RS. Data are shown as mean ± SD. */#*p* < 0.05; **/##*p* < 0.01, by Student’s *t* test

To investigate the roles of HFCS and RS in CRC, we established an AOM/DSS-induced colon carcinogenesis model in vivo. We administrated PBS, limited HFCS, or limited HFCS with RS to AOM/DSS-treated C57BL/6 mice for 2 months in specific pathogen-free (SPF) facilities (Fig. 1A). During the modeling stage, we monitored body weight, and there was no significant obesity difference between the three groups (Fig. 1B, supplementary Fig. 1A). In three cycles of DSS treatment, we observed that mice with HFCS intervention showed the highest disease activity index (DAI) scores, while RS could relieve it (Fig. 1C, supplementary Fig. 1B). After 2 months of treatment, the colons were surgically excised. Data from the gross images and H&E staining of colons showed that HFCS promoted colon tumorigenesis in AOM/DSS mice, as compared with PBS control (Fig. 1D, E, supplementary Fig. 1C). Intriguingly, RS supplement could obviously suppress HFCS-induced colon tumorigenesis. In addition, colon tumors in mice treated with HFCS exhibited elevated expression of the cell proliferation marker Ki67, whereas administration of RS was able to counteract this tendency (Fig. 1F), which was quantified by histochemistry score (H-score) (Fig. 1G). Taken together, these data suggested that RS could suppress HFCS-induced colon tumorigenesis in AOM/DSS mice.

### RS suppressed HFCS-induced colon tumorigenesis in *Apc*^*Min/*+^ mice

**Fig. 2:**
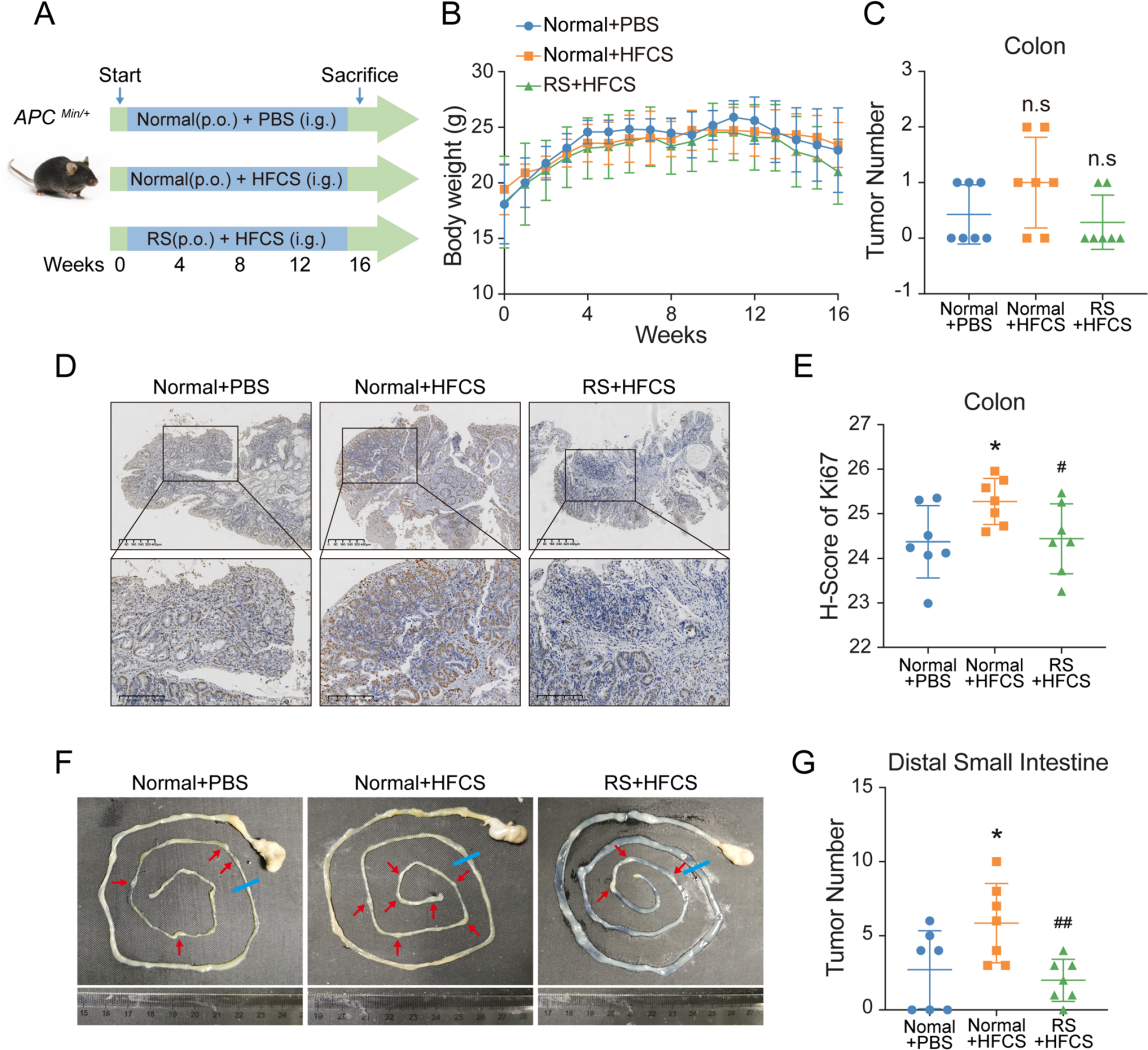
RS suppressed HFCS-induced colon tumorigenesis in *Apc*^*Min/*+^ mice. **A** Schematic illustration of *Apc*^*Min/*+^ mice model in vivo. **B** Body weight of *Apc*^*Min/*+^ mice after the indicated treatment. **C** The number of colon tumors of *Apc*^*Min/*+^ mice treated with HFCS (*n* = 7), RS + HFCS (*n* = 7), or PBS (*n* = 7) as control is quantified. **D**, **E** Representative IHC images of Ki67 protein expression in colon tumor tissues are shown, and the relative expression is quantified by H-score. **F**, **G** Representative small intestines images of *Apc*^*Min/*+^ mice are shown, and the number of tumor per mice is quantified. The red arrows indicate the tumor locations, and the blue line is the cutoff rule of proximal and distal small intestine. *The difference between group PBS and group HFCS. #The difference between group HFCS and group RS + HFCS. Data are shown as mean ± SD. *n.s* no significance; */#*p* < 0.05; ##*p* < 0.01, by Student’s *t* test

In addition to the AOM/DSS model of inflammation-related carcinogenesis, we established an *Apc*^*Min/*+^ mice model, which is a widely used model of spontaneous intestinal adenomas (Fig. 2A). Similarly, we did not find any obesity and metabolic disturbance in mice with defined daily dose HFCS administration when compared to the control (Fig. 2B). Due to the unique feature of *Apc*^*Min/*+^ mice, the number of colorectal tumors was small, almost less than three, and the difference between groups was not statistically significant (Fig. 2C, supplementary Fig. 2A–B). Therefore, we further evaluated the degree of cell proliferation and found that colon tumors from HFCS-treated mice had increased expression of cell proliferation marker Ki67 (Fig. 2D, E). Similarly, RS rescued the increased H-score of Ki67 in HFCS treatment. Moreover, the number of tumors in distal small intestine was counted. The intake of HFCS significantly increased the number of tumors, while the addition of RS inhibited the promotion effect of HFCS (Fig. 2F, G). Taken together, these data suggested that RS also suppressed HFCS-induced colon tumorigenesis in *Apc*^*Min/*+^ mice.

### Intake of HFCS and RS reshaped the microbial community

**Fig. 3:**
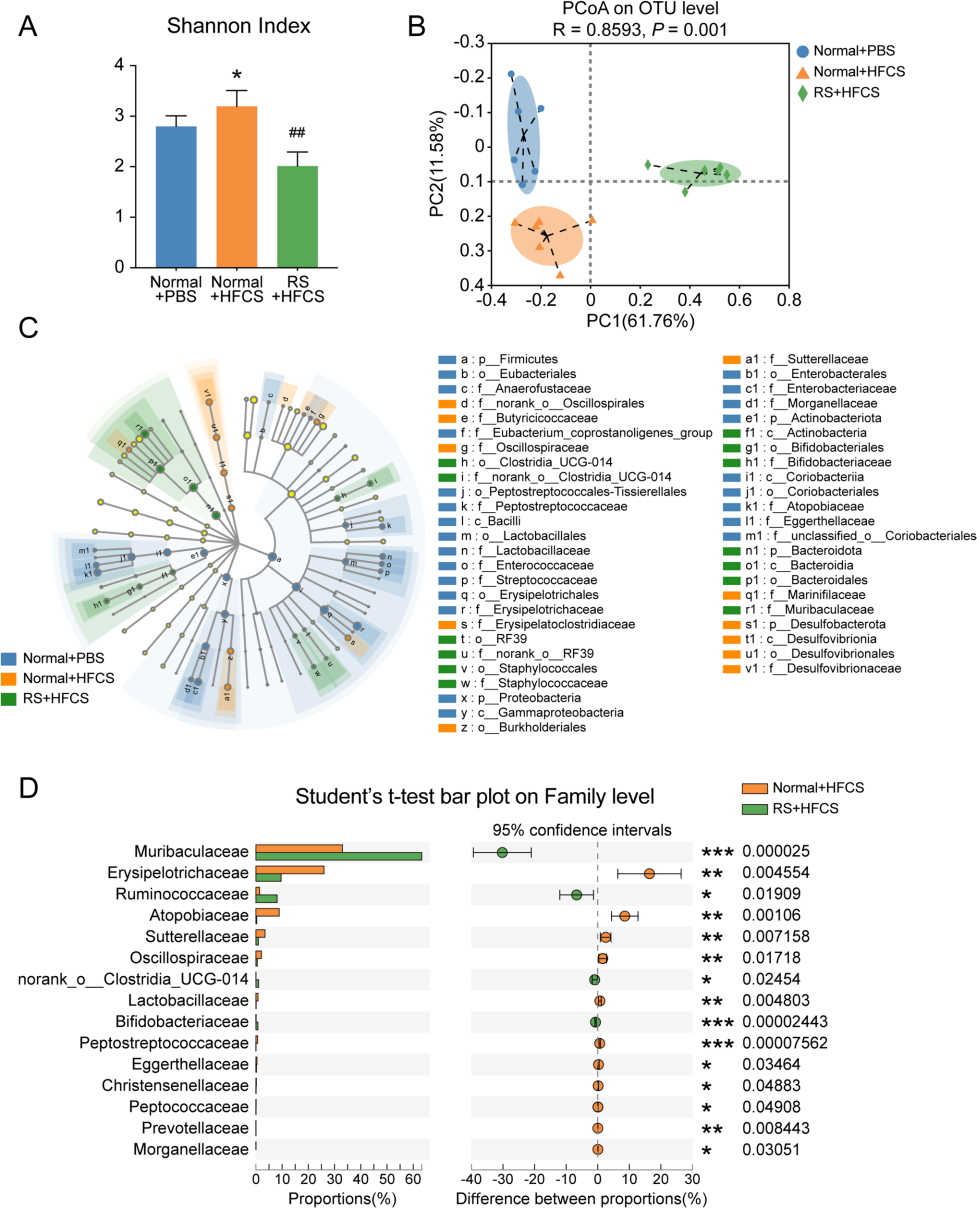
Intake of HFCS and RS reshaped the microbial community. **A** 16S rRNA sequencing was conducted using stool samples collected from AOM/DSS mice. The α-diversity of the gut microbiome was determined by Shannon index. **B** The PCoA plot shows the β-diversity of the gut microbiome. **C** Bacterial taxa identified as differentially abundant among the groups with different treatments according to LEfSe analysis. Different colors indicate that the abundance of bacterial taxa is higher in the indicated group. **D** Significant family alterations in mice treated with HFCS and HFCS with the addition of RS. Data are shown as mean ± SD. *p < 0.05; **/##*p* < 0.01; ***p < 0.001, by Student’s *t* test or Wilcoxon rank-sum test

To understand the underlying mechanisms of RS-induced colon tumorigenesis suppression, we directly incubated RS with CRC cell lines of LoVo and HCT116, as well as human normal colonic epithelial cell line of NCM460 in vitro. CCK-8 assay showed that RS did not influence the cell proliferation ability in different cell lines, even in different concentrations (Supplementary Fig. 3A–C), indicating that RS had no direct effect on colonic epithelium and tumorigenesis. Numerous studies have demonstrated that intestinal microbiota played a critical role in the alleviation or development of CRC [6, 29, 30]. Furthermore, RS diet was reported to affect intestinal microbiota composition [31], and RS-associated gut microbiota variations played a vital role in the therapeutic effects of dietary changes [32, 33]. Therefore, we collected mice stools from AOM/DSS mice for 16S rRNA sequencing to investigate the variations of the microbial community. We found that the diversity within the microbial community was increased after HFCS administration compared to the control as assessed by Shannon index (α-diversity), while RS supplementation reduced this tendency (Fig. 3A). Furthermore, we examined the microbial construction in different treatments by the principal coordinate analysis (PCoA) plot on operational taxonomic units (OUTs) level, which clearly showed the distance of sample groups (β-diversity) (Fig. 3B). Therefore, the results showed that both HFCS and RS administration could cause changes in intestinal flora composition.

To further characterize phenotypic changes in the taxonomic composition, we performed Linear discriminant analysis Effect Size (LEfSe) to identify differentially abundant biomarkers with biological consistency among the three groups (Fig. 3C). Moreover, we compared the relative taxa abundance at family levels using Student’s *t* test. The abundances of *Muribaculaceae* [34] and *Ruminococcaceae* [35, 36], which have been reported as potentially protective probiotics against CRC, were significantly upregulated in RS-treated mice than that in the HFCS group. Conversely, *Erysipelotrichaceae* [37] was notably decreased after RS treatment, and it was demonstrated to enhance cell growth in CRC (Fig. 3D)*.* Interestingly, when we further evaluated the correlation between gut microbiota and tumor loads, we found that the relative abundances of *Muribaculaceae* and *Ruminococcaceae* were both negatively correlated with tumor numbers (Supplementary Fig. 3D–E). There was a positive correlation trend between *Erysipelotrichaceae* and tumor numbers, even with no statistical difference (Supplementary Fig. 3F). Together, these results indicated that the administration of HFCS and RS influenced colon tumorigenesis along with microbial disorder, and gut microbiota might be involved in the anti-tumor effects of RS.

### Intake of HFCS and RS affected the levels of intestinal SCFAs

**Fig. 4:**
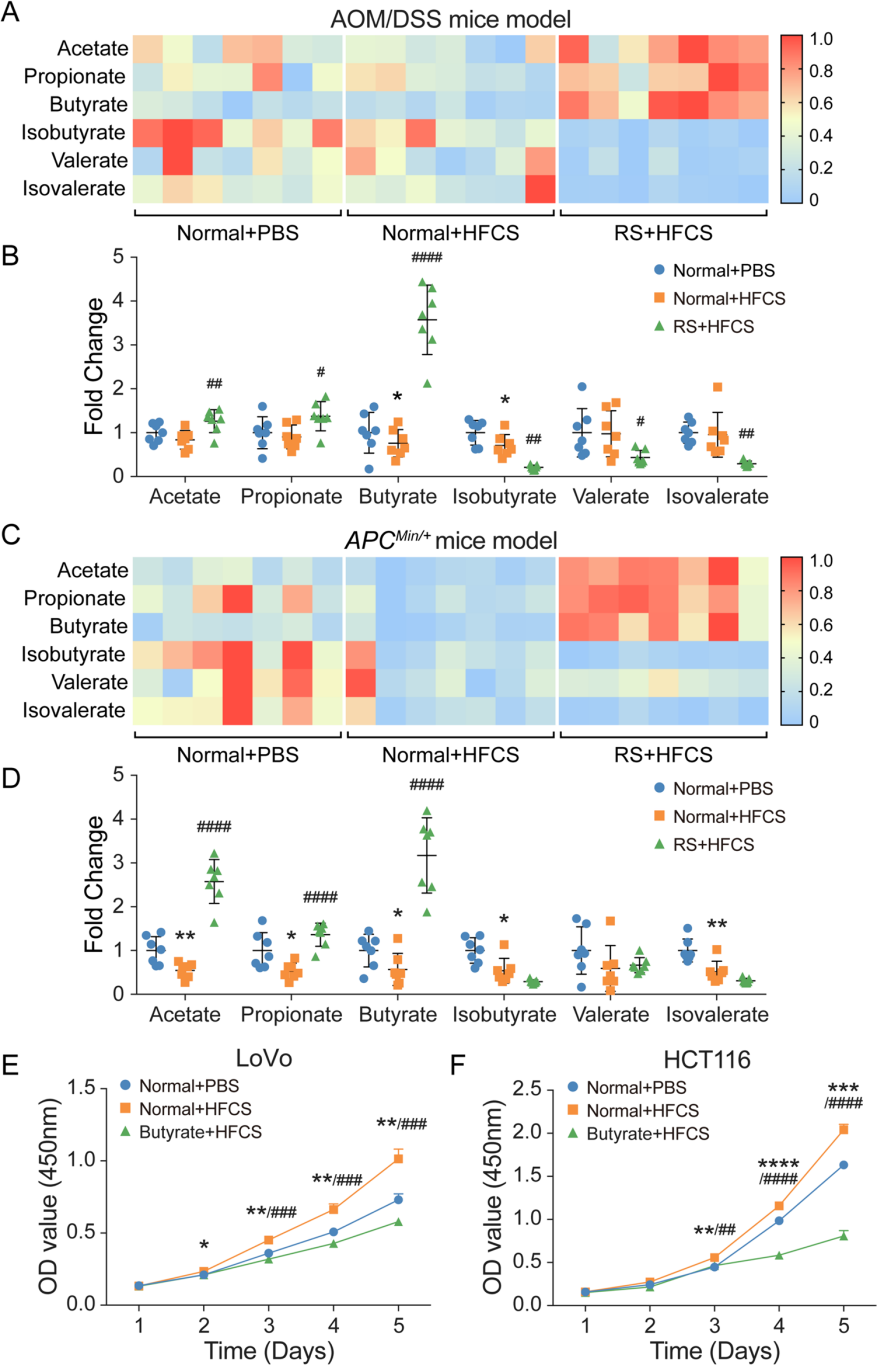
Intake of HFCS and RS affected the levels of intestinal SCFAs. **A**, **B** The relative abundance of acetate, propionate, butyrate, isobutyrate, valerate, and isovalerate in feces of AOM/DSS mice treated with HFCS (*n* = 7), RS + HFCS (*n* = 7), or PBS (*n* = 7) as control, which was measured by gas chromatography. **C**, **D** The relative abundance of acetate, propionate, butyrate, isobutyrate, valerate, and isovalerate in feces of *Apc*^*Min/*+^ mice in different groups. **E**, **F** LoVo and HCT116 cells were co-cultured with HFCS, butyrate + HFCS, or PBS control and subjected to CCK-8 assay. *The difference between group PBS and group HFCS. #The difference between group HFCS and group RS (butyrate) + HFCS. Data are shown as mean ± SD. */#*p* < 0.05; **/##*p* < 0.01; ***/###*p* < 0.001; ****/####*p* < 0.0001, by Student’s *t* test

While it remains unclear how changes in the gut microbiota contribute to benefits in the host, a possible mechanism is through altered metabolic production. Given RS is known as a source of SCFAs production via bacterial fermentation in the colon [38], and RS diet significantly increased the abundance of SCFAs-producing bacteria of *Muribaculaceae* [39] and *Ruminococcaceae* [40], we next evaluated the variations of SCFAs in the intestine by targeted metabolomics. We collected stools from both AOM/DSS and *Apc*^*Min/*+^ mice, and detected the abundance of SCFAs by gas chromatography assay. In the AOM/DSS-induced colon carcinogenesis model, the administration of HFCS could obviously suppress the levels of butyrate compared to the control, with a similar change tendency of acetate, propionate, isobutyrate, valerate, and isovalerate. However, the supplementation of RS increased the production of SCFAs, especially the levels of butyrate and acetate, which was consistent with the previous findings [41, 42] (Fig. 4A, B). Similarly, we evaluated the alterations of SCFAs in *Apc*^*Min/*+^ mice model, and the results showed consistent trends (Fig. 4C, D).

In addition, to further evaluate the vital role of microbe-derived metabolite SCFAs in the anti-tumor effect of RS, we established an antibiotics-induced microbiota depletion mice model. C57BL/6 mice with subcutaneous inoculation of CRC cells were fed with either RS or control diet, along with or without antibiotics cocktail treatment as previously reported [27] (Supplementary Fig. 4A). As expected, the administration of RS could obviously increase the levels of SCFAs compared to the control, whereas gut microbiota depletion by antibiotics presented the opposite effect (Supplementary Fig. 4B). Furthermore, we found that tumor-suppressive effect of RS on MC38 subcutaneous tumors was abrogated by antibiotics cocktail treatment (Supplementary Fig. 4C–F).

Further, we tested whether butyrate or acetate affected CRC cell proliferation in vitro. As the data showed, butyrate significantly suppressed cell proliferation in a concentration-dependent manner (Supplementary Fig. 5A–B). Similar results were also observed in acetate treatment (Supplementary Fig. 5C–D). Importantly, butyrate could obviously inhibit cell proliferation even at a lower concentration compared to that with acetate treatment. Therefore, it is plausible that the abundance of SCFAs, especially butyrate, may underlie the anti-tumor effect of RS in HFCS-induced colon tumorigenesis. To further verify our hypothesis, we added HFCS with butyrate to the culture medium of LoVo and HCT116 cells, and performed CCK-8 assay. The results showed that HFCS significantly facilitated CRC cell proliferation, while the addition of butyrate rescued the promoting effects (Fig. 4E, F). Taken together, our results indicated the potential critical roles of microbe-derived metabolite SCFAs, especially butyrate, in the regulation of CRC in HFCS and RS administration.

### Intake of RS inhibited the promotion of glycolysis by HFCS in CRC

**Fig. 5:**
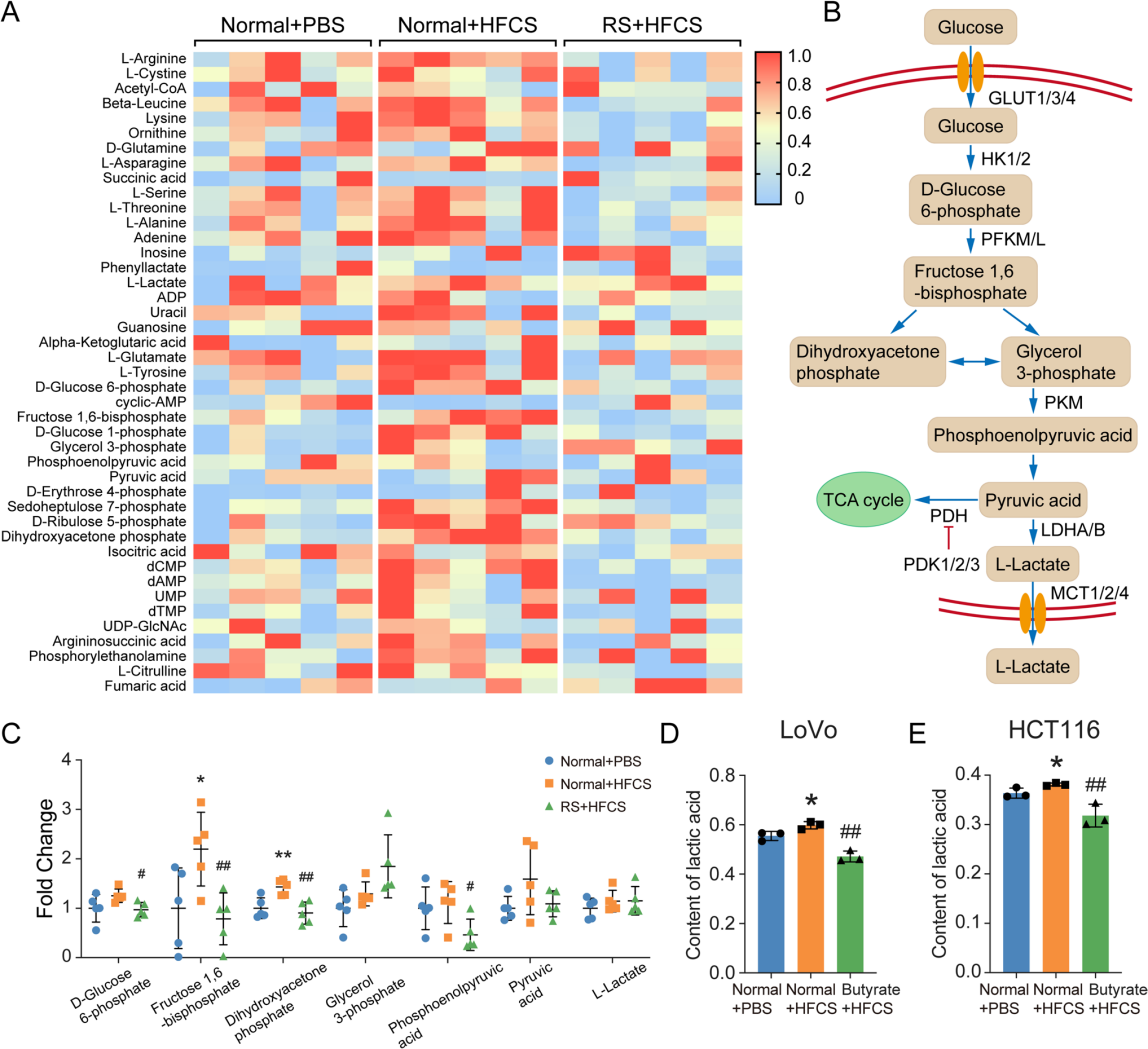
Intake of RS inhibited the promotion of glycolysis by HFCS in CRC. **A** The glycolysis levels of colon tumors in AOM/DSS mice were detected by mass spectrometry analysis. **B** Schematic illustration of key enzymes and metabolites in the glycolysis process. **C** The relative abundance of D-glucose 6-phosphate, fructose 1,6-bisphosphate, dihydroxyacetone phosphate, glycerol 3-phosphate, pyruvic acid, and L-lactate in colon tumor tissues of mice with different treatments. **D, E** The levels of lactic acid in the cell supernatant of LoVo and HCT116 cells with the indicated treatment were measured. *The difference between group PBS and group HFCS. #The difference between group HFCS and group RS (butyrate) + HFCS. Data are shown as mean ± SD. */#*p* < 0.05; **/##*p* < 0.01, by Student’s *t* test

It was reported that HFCS could facilitate intestinal tumorigenesis in mice by accelerating glycolysis [12]. Therefore, we next investigated the glycolysis levels of colon tumors from AOM/DSS mice, assessing the abundance of glycolytic intermediates and end products by mass spectrometry analysis (Fig. 5A). The results showed that HFCS treatment enhanced glycolysis, as reflected by the increased levels of D-glucose 6-phosphate, fructose 1,6-bisphosphate, dihydroxyacetone phosphate, glycerol 3-phosphate, pyruvic acid, and L-lactate (Fig. 5B, C). Among all the changed glycolytic intermediates, the alteration of fructose 1,6-bisphosphate was the most statistically significant, which was reported to be the key factor of glycolysis activation in supporting tumor growth [12]. Remarkably, the addition of RS reverses the promotion of glycolysis level, indicating the important roles of glycolysis in the RS-mediated colon tumorigenesis suppression. Since butyrate mediates the functions of RS, we treated LoVo and HCT116 cells with HFCS, HFCS with butyrate, or PBS control and detected the lactic acid levels in the cell supernatant. Consistent with the observation in vivo, HFCS treatment enhanced lactate production, and the promotion effect was attenuated after the addition of butyrate in vitro (Fig. 5D, E). Taken together, our results revealed that the intake of RS inhibited the promotion of glycolysis by HFCS in CRC.

### Butyrate suppressed glycolysis and CRC cell proliferation by downregulating HK2

**Fig. 6:**
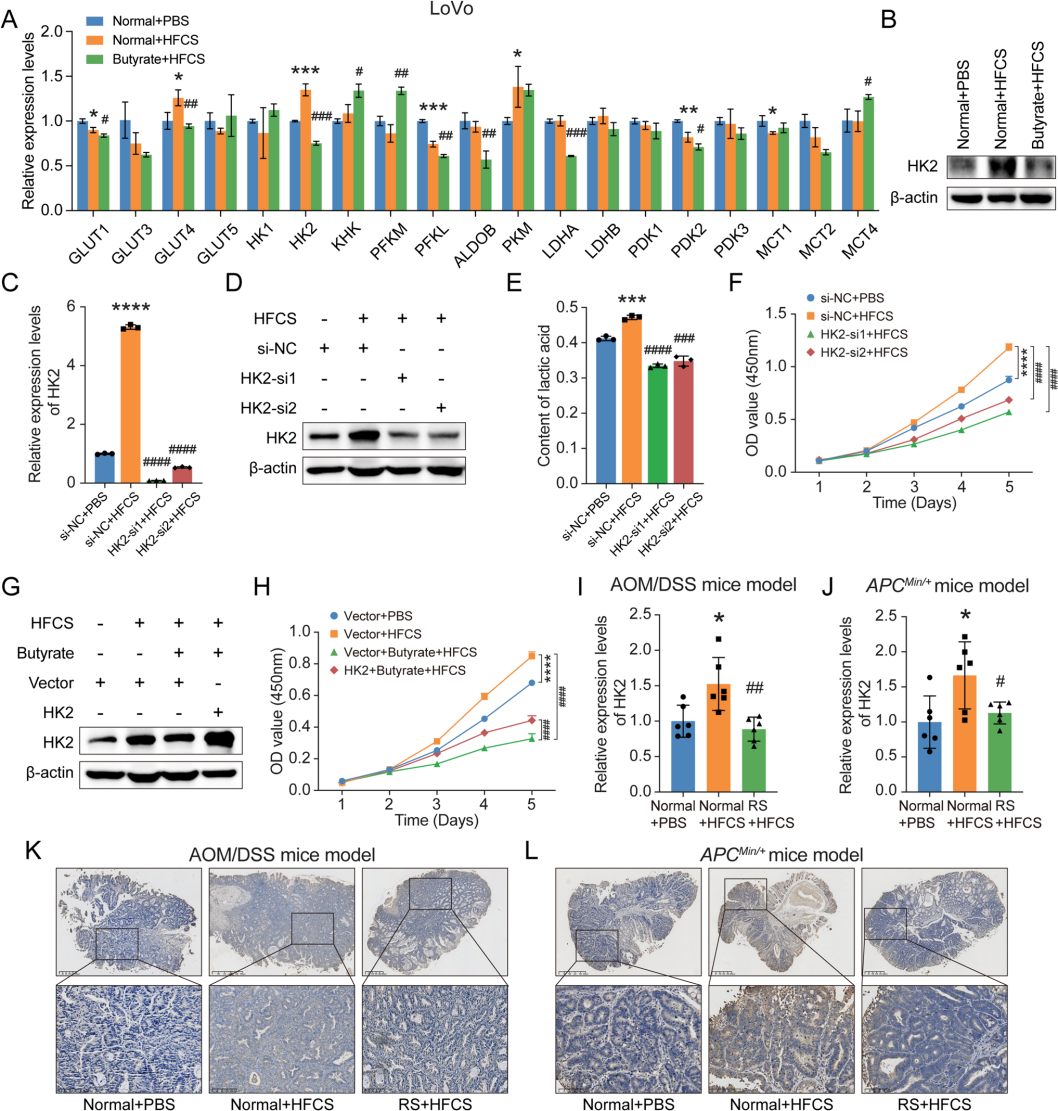
Butyrate suppressed glycolysis and CRC cell proliferation by downregulating HK2. **A** The relative expression levels of the key enzymes of glycolysis in LoVo cells treated with HFCS, butyrate + HFCS, or PBS control. **B** Western blot analysis of HK2 was performed in LoVo cells with the indicated treatment. **C**, **D** Quantitative RT-PCR and western blot analysis of HK2 were performed in LoVo cells. They were transfected with two siRNAs targeting HK2 or control siRNAs, and then co-cultured with HFCS or PBS control. **E**, **F** LoVo cells with the indicated treatment were subjected to lactic acid detection analysis and CCK-8 assay. **G**, **H** LoVo cells transfected with the indicated plasmids were co-cultured with HFCS, butyrate + HFCS or PBS control, and subjected to western blot analysis and CCK-8 assay. **I**, **J** Quantitative RT-PCR of the relative mRNA levels of HK2 in colon tumor tissues from mice with the indicated treatment was performed. **K**, **L** IHC staining of HK2 in colon tumor tissues from the indicated mice. Data are shown as mean ± SD. */#*p* < 0.05; **/##*p* < 0.01, ***/###*p* < 0.001, ****/####*p* < 0.0001, by Student’s *t* test

Since butyrate underlies the anti-tumor effect of RS in HFCS-induced colon tumorigenesis, and butyrate suppresses the enhanced glycolysis induced by HFCS, we further screened for key glycolytic enzymes variations upon butyrate treatment in vitro. Quantitative RT-PCR results revealed a significant upregulation of HK2 in LoVo cells with HFCS treatment compared with PBS control, and butyrate abrogated this tendency (Fig. 6A). Similarly, western blot analysis also confirmed the variations of HK2 (Fig. 6B). HK2, a predominant isoform of hexokinase, catalyzes the rate-limiting step of phosphorylation of glucose to generate glucose 6-phosphate during glycolysis [43], as shown in the schematic illustration of glycolysis in Fig. 5B. The increased aerobic glycolysis, or the Warburg effect, is another hallmark of cancer [44], and the glycolysis level of tumor cells largely determines their proliferation ability. Previous studies have reported that HK2 is upregulated in many types of tumors associated with enhanced aerobic glycolysis in tumor cells, including CRC [45, 46].

To further investigate whether HK2-mediated glycolysis levels were involved in the enhanced proliferation ability of CRC cells induced by HFCS, we performed HK2 loss of function assays in CRC cells. The upregulated mRNA and protein levels of HK2 stimulated by HFCS were significantly decreased when we silenced the expression of HK2 by two different siRNAs in LoVo cells (Fig. 6C, D) and HCT116 cells (Supplementary Fig. 6A–B). As expected, HK2 knockdown inhibited HFCS-induced glycolysis enhancement and CRC cell proliferation (Fig. 6E–F, supplementary Fig. 6C–D). In addition, both quantitative RT-PCR and western blot analysis showed that RS did not directly affect the expression levels of HK2 in vitro (Supplementary Fig. 6E–F). Therefore, we reintroduced HK2 levels in butyrate-treated CRC cells and found that ectopic expression of HK2 partially reversed the suppressive effects of butyrate on cell proliferation in LoVo cells (Fig. 6G, H) and HCT116 cells (Supplementary Fig. 6G–H).

Moreover, we also detected the expression of HK2 in colon tumor tissues in AOM/DSS and *Apc*^*Min/*+^ mice. Consistently, the mRNA levels of HK2 were significantly higher in mice with HFCS administration, and RS supplement suppressed the upregulation of HK2 in both the mice models (Fig. 6I, J). Furthermore, this observation was also confirmed by immunohistochemistry (IHC) staining analysis on protein levels (Fig. 6K, L). These data indicated that butyrate suppressed glycolysis and CRC cell proliferation by downregulating HK2.

## Discussion

Daily diets have been documented to be involved in disease progression, as well as in CRC [47]. However, the underlying mechanisms still remain elusive. Here, our work revealed that RS suppressed HFCS-induced colon tumorigenesis in both AOM/DSS and *Apc*^*Min/*+^ mice models through reshaping the microbial community. Mechanistically, the alteration of the microbial community after RS supplement increased the levels of intestinal SCFAs, especially butyrate, leading to the suppression of glycolysis and CRC cell proliferation by downregulating HK2.

SSBs are popular foods in our daily life, and they are primarily sweetened with HFCS, which consists of glucose and fructose in a 45:55 ratio [48]. The increased consumption of SSBs has been paralleled by an epidemic of obesity around the world [49, 50]. Actually, studies have shown that excessive intake of SSBs could cause obesity, and obesity would increase the risk of CRC, especially in men [51, 52]. However, due to the two important confounders of obesity and metabolic syndrome, whether SSBs contribute directly to tumorigenesis is unclear. In our study, we administrated limited HFCS to mice and monitored body weight and did not find any obesity and metabolic disturbance when compared to the control. Howecver, a greater number of colon tumors of mice after HFCS treatment were still detected. Consistently, a recent study also confirmed the tumor promotion effects of HFCS in the absence of obesity and metabolic syndrome [12]. Excessive intake of HFCS promotes CRC by inducing metabolic disorder, while constant intake also induces tumorigenesis by some other mechanisms. With the wide application of HFCS, we need to find a suitable way to reduce or inhibit its harmful effects.

RS, a type of dietary fiber, has been extensively studied for the past few decades and found to confer a broad range of health benefits, including the total amount of starch and the products of starch degradation that resist digestion in the small intestine [15]. RS can be classified into four types, type 1 to type 4 according to its properties [16], some of which occur naturally in foods such as potatoes and grains, and some of which are produced or modified commercially. It is reported that engineered RS diet reshaped colon microbiota profile in parallel with the suppression of pancreatic cancer growth in in vitro and in vivo models [53]. Additionally, high levels of RS in diet modulated a specific pattern of miRNAs expression profile, which was associated with a better overall survival in pancreatic cancer [54]. Moreover, dietary type 3 RS prevents colon carcinogenesis in 1,2-dimethylhydrazine-treated Sprague–Dawley rats model, which was reflected by altering proliferation, apoptosis, and dedifferentiation in the rat colon [55]. A randomized controlled trial also revealed that oral supplementation of RS for 4 weeks in patients with CRC reduced the cell cycle regulatory genes CDK4 and GADD45A, inhibiting cell proliferation in the upper part of colonic crypts [24]. Furthermore, Karen Humphreys et al*.* [25] found that red meat and RS had opposite effects on the CRC-promoting microRNAs. We thus hypothesized that the consumption of RS may have an anti-tumor effect in HFCS-related CRC. To verify our hypothesis, we established AOM/DSS and *Apc*^*Min/*+^ mice models in vivo, and administered type 2 RS to HFCS-treated mice in their daily diet. Indeed, we observed that RS significantly suppressed HFCS-induced colon tumorigenesis.

Furthermore, we attempted to reveal the mechanisms for the suppression of CRC by RS. It is generally acknowledged that RS alters the microbial community, and RS-associated gut microbiota variations play a critical role in the therapeutic effects of dietary changes [16, 53, 56–58]. Therefore, we collected mice stools in AOM/DSS models for 16S rRNA sequencing and found that the diversity within the microbial community was decreased after the supplement of RS in HFCS-treated mice (α-diversity). Similarly, β-diversity was also significantly changed, which reflected the microbial community difference among groups. In detail of the changed microbiota taxa, we found that the addition of RS could alleviate the changed microbiota induced by HFCS, and the altered microbiota for example of *Muribaculaceae* and *Ruminococcaceae* [35, 39, 40, 59] were reported to participate in the production of intestinal SCFAs. Furthermore, we established an antibiotics-induced gut microbiota depletion mice model in vivo, and the results confirmed that gut microbiota, at least partially, played an essential role in mediating RS-associated CRC development.

In recent years, several lines of evidence have suggested that the gut microbiota is able to produce or transform a series of metabolites and molecules, including well-established metabolites (i.e., SCFAs, bile acids, trimethylamine N-oxide) and some recently identified molecular actors (i.e., endocannabinoids, bioactive lipids, phenolic-derived compounds, advanced glycation end products, and enterosynes) [60]. Accumulating evidence suggests that RS is a source of SCFAs production via bacterial fermentation in the colon [38]. Interestingly, when we collected stools of different mice models and performed targeted metabolomics, we found that the administration of HFCS and RS obviously affected the levels of intestinal SCFAs. In detail, compared with HFCS-treated mice, the addition of RS increased the production of SCFAs, such as butyrate, acetate, and propionate. Consistently, several previous studies also confirmed the upregulation of SCFAs in the intestine after RS treatment [15, 16, 20]. Gut microbiota depletion experiment in vivo further confirmed the critical role of microbe-derived metabolite SCFAs in the anti-tumor effect of RS. It was reported that acetate and propionate were more likely to be absorbed into the blood, which had a systemic effect on metabolic syndrome [61]. Butyrate was reported to maintain mucosal integrity and suppress inflammation and carcinogenesis through effects on immunity, gene expression, and epigenetic modulation [61].

It is widely accepted that normal differentiated cells mainly rely on oxidative phosphorylation of mitochondria to provide energy for cells, while most tumor cells rely on aerobic glycolysis [44, 62, 63]. The glycolysis level of tumor cells largely determines their proliferation ability. Our results based on mass spectrometry analysis suggested that HFCS treatment enhanced glycolysis in the colon tumors of AOM/DSS mice, which was consistent with the previous findings by Marcus D. Goncalves [12]. Therefore, we wondered whether RS could play an anti-tumor role by inhibiting aerobic glycolysis. Remarkably, the administration of RS was found to counteract the promotion of glycolysis levels, as reflected by decreased D-glucose 6-phosphate, fructose 1,6-bisphosphate, dihydroxyacetone phosphate, glycerol 3-phosphate, pyruvic acid, and L-lactate. However, maybe due to the sample size, the difference was not statistically significant, even with a trend. It will be of great interest to expand the sample size in our future research.

Furthermore, we attempted to uncover whether butyrate affects aerobic glycolysis in CRC cells. Consistent with the observation in vivo, HFCS treatment enhanced lactate production, and the promotion effect was attenuated after the addition of butyrate, indicating the inhibitory effect of butyrate on glycolysis. As is known, hexokinases catalyze the first committed step of glucose metabolism by phosphorylating glucose to glucose-6-phosphatase [43]. HK2, one of the major hexokinase isoforms, is critically important for aerobic glycolysis in multiple cancer types, including CRC [45, 46], hepatocellular carcinoma [64], glioblastoma multiforme [65], breast cancer [66], ovarian cancer [67], etc. In our study, HFCS increased the levels of HK2 in CRC cells, and butyrate supplement significantly abrogated this tendency. We found that HK2, at least partially, mediated the functions of RS in suppressing glycolysis and CRC cell proliferation in vitro. In addition, the expression of HK2 was significantly higher in mice with HFCS administration, and the addition of RS obviously suppressed the promotion effects. Collectively, our results suggest that RS, mainly depends on microbe-derived metabolite SCFAs of butyrate, suppresses glycolysis and CRC cell proliferation by downregulating HK2 during colorectal carcinogenesis.

Despite the findings, this study still has some limitations. Firstly, gut microbiota depletion is only one of the common methods to explain the role of gut microbiota; fecal microbiota transplantation or germ-free mice could also be applied, which require more study. Secondly, we only focused on SCFAs, well-known metabolites of RS for the mechanistic investigations, and other bioactive substances might also contribute to the effects of RS, which require further study by untargeted metabolomics analyses. Further research may reveal other possible molecular mechanisms by which the RS-altered metabolites or gut microbes lead to the inhibition of colorectal cancer. Finally, the experiments were performed using murine models and colorectal cancer cell lines, and the conclusions of our study need further validation in clinical research.

In summary, our current findings provide important insights into the potential mechanisms underlying diets containing HFCS and RS in CRC. RS alters the microbial community, resulting in increased levels of intestinal SCFAs, and suppressed glycolysis and colon tumorigenesis by downregulating HK2. Since the compelling evidences in vitro and in vivo highlight the emergence of RS as a functional food benefit to the colorectum, a potential therapeutic strategy targeting RS to antagonize the adverse effect of HFCS could be well utilized.

## Supplementary Information

Below is the link to the electronic supplementary material.

Supplementary file1 (DOCX 135616 KB)

Supplementary file2 (DOCX 13 KB)
