# Supplementary material for: Resistant starch reduces glycolysis by HK2 and suppresses high-fructose corn syrup-induced colon tumorigenesis
Source: J Gastroenterol. 2024 Aug 14;59(10):905–20. doi: 10.1007/s00535-024-02138-3 (PMC11415400; doi:10.1007/s00535-024-02138-3)
Supplement: Supplementary file 1 — Supplementary file1 (DOCX 135616 KB) [file 535_2024_2138_MOESM1_ESM.docx]

**Supplementary Figures**

**
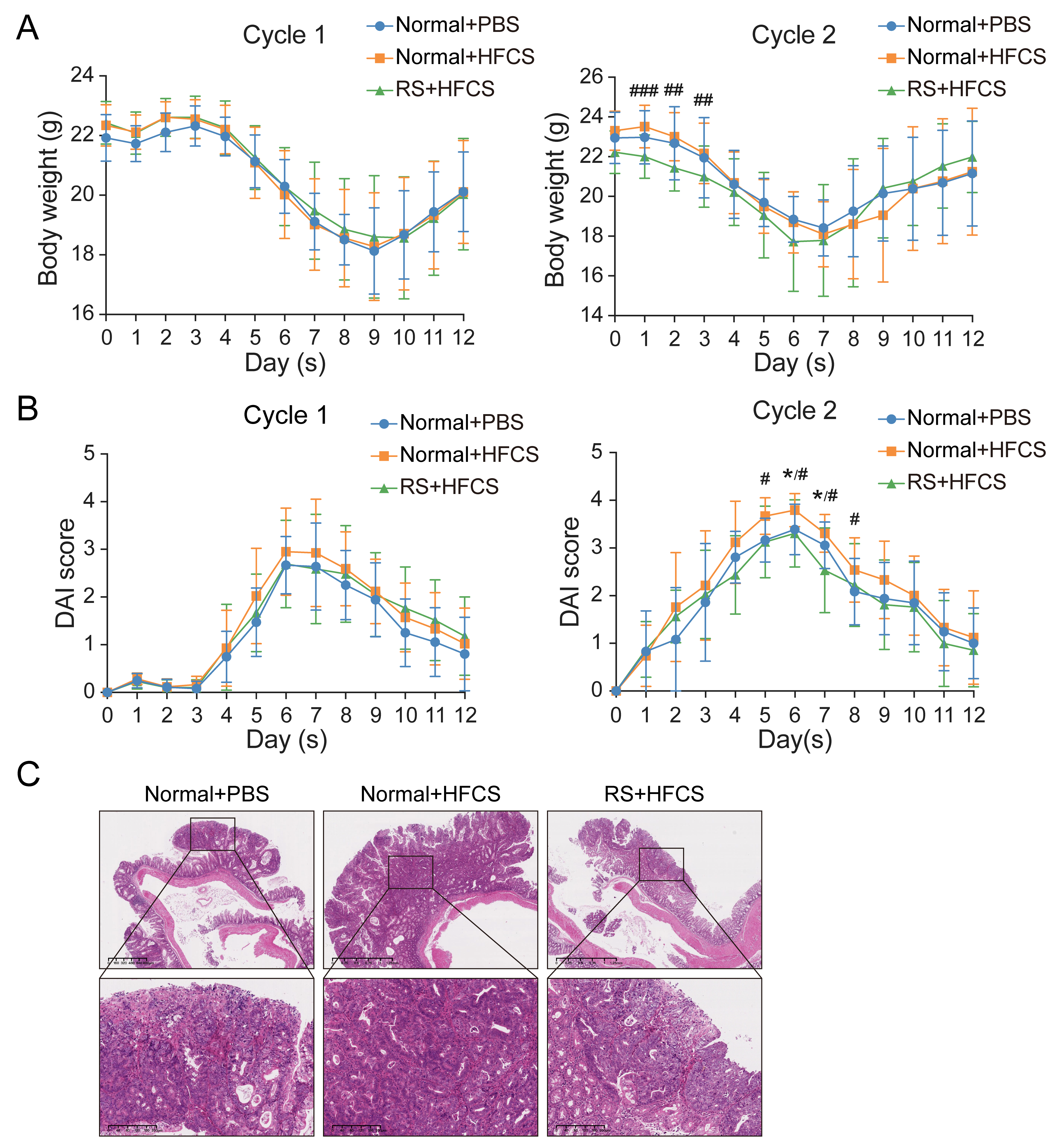
Supplementary Figure 1. RS suppressed HFCS-induced colon tumorigenesis in AOM/DSS mice.** (A-B) Body weight (A) and DAI scores (B) of AOM/DSS mice in cycle 1 and cycle 2 of DSS administration. (C) Representative H&E images of colon tumor tissues from the indicated mice were shown. Data are shown as mean ± SD. */#, p < 0.05, by Student’s t test.

**
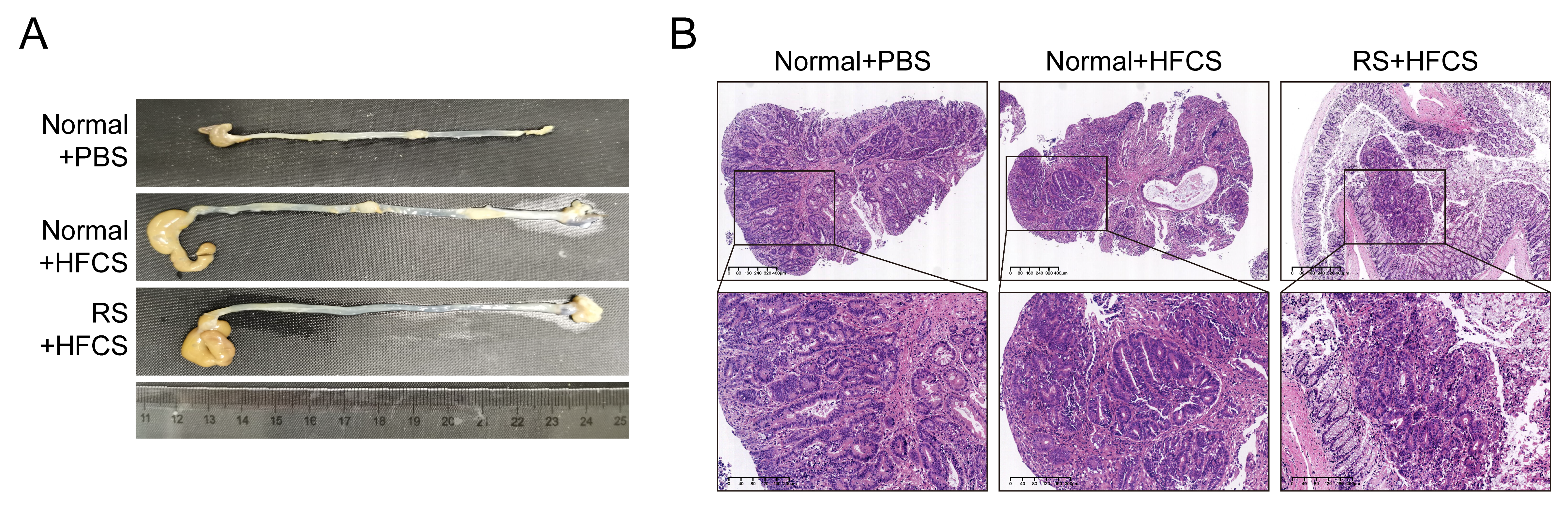
Supplementary Figure 2. RS suppressed HFCS-induced colon tumorigenesis in *Apc^Min/+^* mice.** (A) Representative colon images of *Apc^Min/+^* mice were shown. (B) Representative H&E images of colon tumor tissues from the indicated mice were shown.


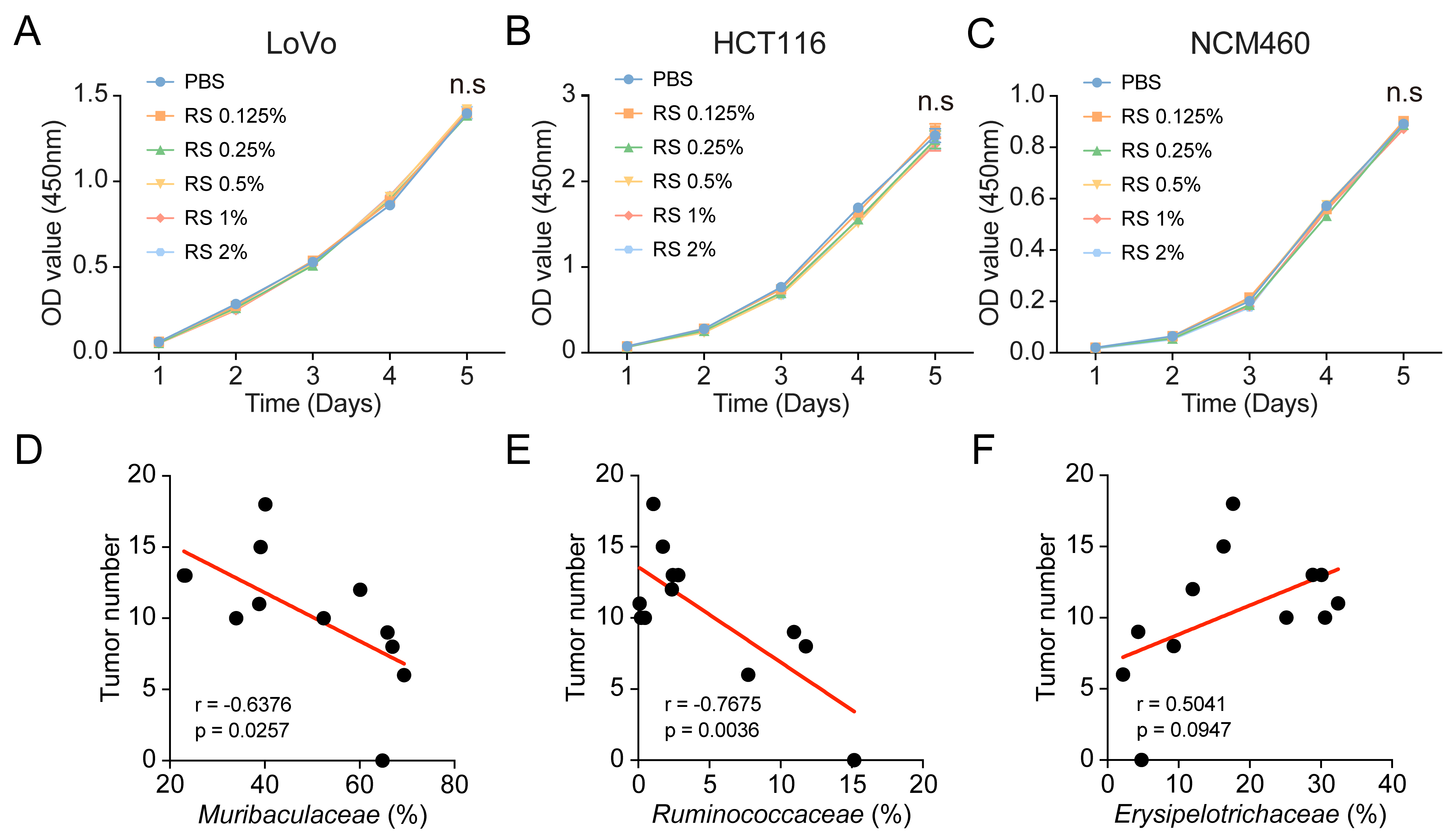


**Supplementary Figure 3. RS had no direct effect on colonic epithelium and tumorigenesis.** (A-C) LoVo, HCT116 and NCM460 cells were co-cultured with different concentrations of RS (0.125%, 0.25%, 0.5%, 1%, 2%) or PBS control, and subjected to CCK-8 assay. (D-F) Pearson r values between the relative abundance of *Muribaculaceae* (D), *Ruminococcaceae* (E), *Erysipelotrichaceae* (F), and tumor numbers in AOM/DSS mice. Data are shown as mean±SD. n.s, no significance, by Student’s t test (A-C), and Linear Regression (D-F).


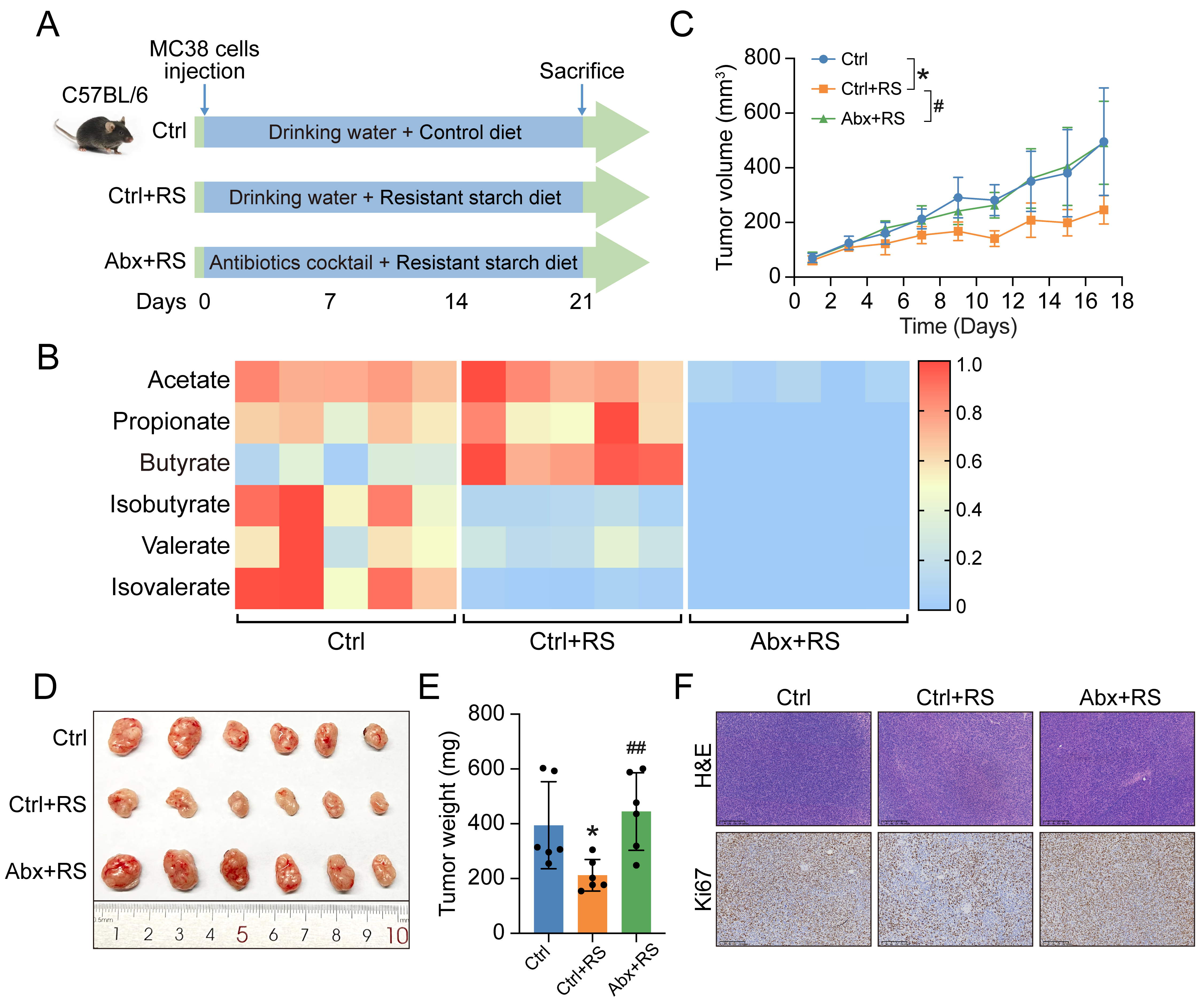


**Supplementary Figure 4. Microbe-derived metabolites SCFAs mediated the anti-tumor effect of RS *in vivo*.** (A) Schematic illustration of subcutaneous tumor model *in vivo*. (B) The relative abundance of acetate, propionate, butyrate, isobutyrate, valerate and isovalerate in feces of mice in subcutaneous tumor model, which was measured by gas chromatography. (C) Tumor volume of control-fed (n = 6), RS-fed (n = 6), and Abx-treated RS-fed (n = 6) mice before sacrifice. (D-E) Gross images and tumor weight of subcutaneous tumors at sacrifice. (F) Representative images of H&E staining and IHC staining for Ki67 in subcutaneous tumor tissues. Black scale bars, 200 µm. *, the difference between control-fed and RS-fed. #, the difference between RS-fed and Abx-treated RS-fed. Data are shown as mean±SD. n.s, no significance,*/#, p < 0.05; ##, p < 0.01, by Student’s t test.


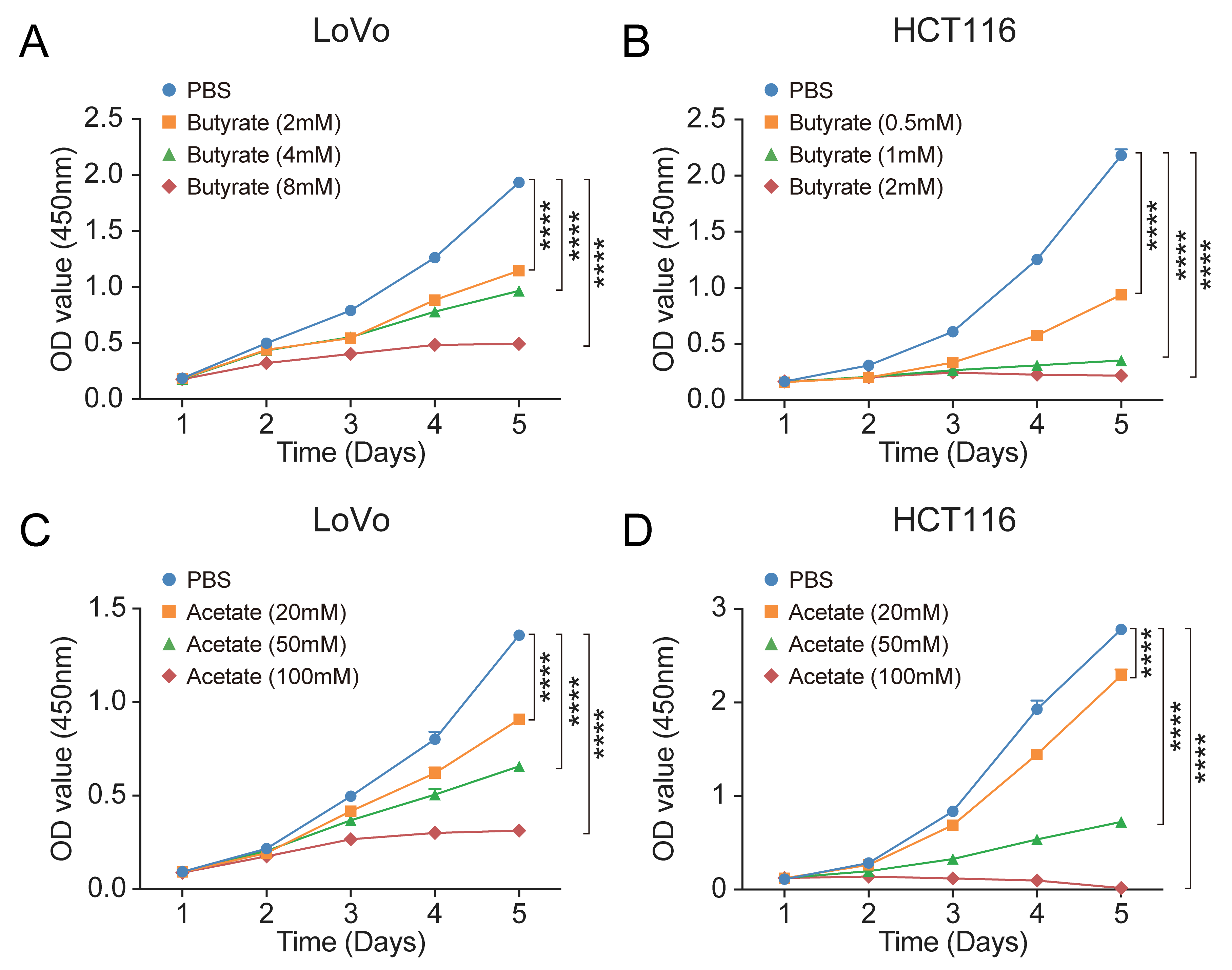


**Supplementary Figure 5. Both butyrate and acetate suppressed CRC cell proliferation.** (A-B) LoVo and HCT116 cells were treated with butyrate in different concentrations (2mM, 4mM, 8mM; 0.5mM, 1mM, 2mM), and subjected to CCK-8 assay. (C-D) LoVo and HCT116 cells were treated with acetate in different concentrations (20mM, 50mM, 100mM; 20mM, 50mM, 100mM), and subjected to CCK-8 assay. Data are shown as mean ± SD. ****, p < 0.0001, by Student’s t test.


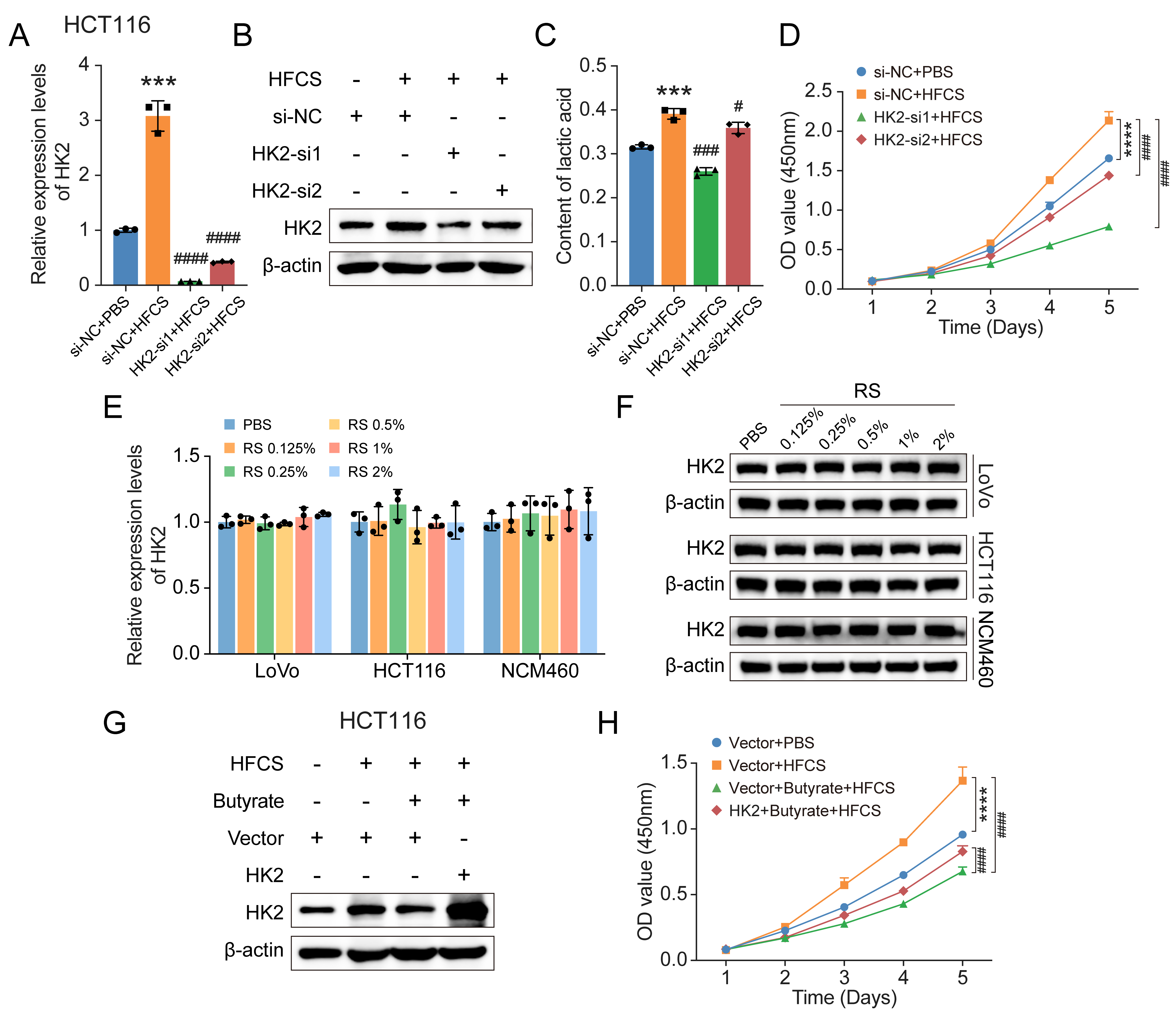


**Supplementary Figure 6. HK2 was involved in the anti-tumor mechanisms of RS.** (A-B) Quantitative RT-PCR (A) and western blot analysis (B) of HK2 were performed in HCT116 cells. They were transfected with two siRNAs targeting HK2 or control siRNAs, and then co-cultured with HFCS or PBS control. (C-D) HCT116 cells with the indicated treatment were subjected to lactic acid detection analysis (C) and CCK-8 assay (D). (E-F) LoVo, HCT116 and NCM460 cells were co-cultured with different concentrations of RS (0.125%, 0.25%, 0.5%, 1%, 2%) or PBS control, and subjected to quantitative RT-PCR (E) and western blot analysis (F). (G-H) HCT116 cells transfected with the indicated plasmids were co-cultured with HFCS, butyrate + HFCS or PBS control, and subjected to western blot analysis (G) and CCK-8 assay (H). Data are shown as mean ± SD. #, p < 0.05; ***/###, p < 0.001; ****/####, p < 0.0001, by Student’s t test.
