# Supplementary material for: Resistant starch reduces glycolysis by HK2 and suppresses high-fructose corn syrup-induced colon tumorigenesis
Source: J Gastroenterol. 2024 Aug 14;59(10):905–20. doi: 10.1007/s00535-024-02138-3 (PMC11415400; doi:10.1007/s00535-024-02138-3)
Supplement: Supplementary file 2 — Supplementary file2 (DOCX 13 KB) [file 535_2024_2138_MOESM2_ESM.docx]

**Supplementary Table 1. Primer sequences for qRT-PCR used in this study**

| **Primer Names** | **Sequences** |
| --- | --- |
| Human *β-actin* Forward | AGAGCTACGAGCTGCCTGAC |
| Human *β-actin* Reverse | AGCACTGTGTTGGCGTACAG |
| Human *GLUT1* Forward | TCTGGCATCAACGCTGTCTTC |
| Human *GLUT1* Reverse | CGATACCGGAGCCAATGGT |
| Human *GLUT3* Forward | GAGGACGTGGAGAAAACTTGC |
| Human *GLUT3* Reverse | AGCCGATTGTAGCAACTGTGA |
| Human *GLUT4* Forward | ATCCTTGGACGATTCCTCATTGG |
| Human *GLUT4* Reverse | CAGGTGAGTGGGAGCAATCT |
| Human *GLUT5* Forward | ACGTTGCTGTGGTCTGTAACC |
| Human *GLUT5* Reverse | CATTAAGATCGCAGGCACGATA |
| Human *HK1* Forward | CACATGGAGTCCGAGGTTTATG |
| Human *HK1* Reverse | CGTGAATCCCACAGGTAACTTC |
| Human *HK2* Forward | TTGACCAGGAGATTGACATGGG |
| Human *HK2* Reverse | CAACCGCATCAGGACCTCA |
| Human *KHK* Forward | CAGCGGATAGACGCACACAA |
| Human *KHK* Reverse | GCCACATCTTTGCTGACAAAC |
| Human *PFKM* Forward | TGGAGATGCCCAAGGTATGAAT |
| Human *PFKM* Reverse | GGAGTCGTCCTTCTCGTTCC |
| Human *PFKL* Forward | CCTCACAGGTGCCAACATCT |
| Human *PFKL* Reverse | TCGATGACCTCCATGATGCG |
| Human *ALDOB* Forward | TGTCTGGTGGCATGAGTGAAG |
| Human *ALDOB* Reverse | GGCCCGTCCATAAGAGAAACTT |
| Human *PKM* Forward | ATGTCGAAGCCCCATAGTGAA |
| Human *PKM* Reverse | TGGGTGGTGAATCAATGTCCA |
| Human *LDHA* Forward | TTGACCTACGTGGCTTGGAAG |
| Human *LDHA* Reverse | GGTAACGGAATCGGGCTGAAT |
| Human *LDHB* Forward | CAGGCCCTACTTGTCCTTGT |
| Human *LDHB* Reverse | CAAGTTCATCAGCCAGAGACTTTC |
| Human *PDK1* Forward | GAGAGCCACTATGGAACACCA |
| Human *PDK1* Reverse | GGAGGTCTCAACACGAGGT |
| Human *PDK2* Forward | CCACAACCAAAGTCGCGG |
| Human *PDK2* Reverse | GCATTGCTGGATCCGAAGTC |
| Human *PDK3* Forward | GCTGTCATTTATTTGAAGGCTCT |
| Human *PDK3* Reverse | CTCAGAGCAGGTGGGAGAAA |
| Human *MCT1* Forward | TGGAGTCATTGGAGGTCTTGG |
| Human *MCT1* Reverse | CACAGCAGTTTAGTAGCAAGCC |
| Human *MCT2* Forward | GCCAGAGACCAGATAAAGAGTCA |
| Human *MCT2* Reverse | TCCAACCCCATCCTCCATCT |
| Human *MCT4* Forward | CCATGCTCTACGGGACAGG |
| Human *MCT4* Reverse | GCTTGCTGAAGTAGCGGTT |
| Mouse *β-actin* Forward | GAGACCTTCAACACCCCAGC |
| Mouse *β-actin* Reverse | GGAGAGCATAGCCCTCGTAGAT |
| Mouse *Hk2* Forward | CTACCCGGAGTTGTTCTGCTT |
| Mouse *Hk2* Reverse | CTCACTGGGTCACTAAGGCTC |
